# Prenatal Organophosphates Exposure Alternates the Cleavage Plane Orientation of Apical Neural Progenitor in Developing Neocortex

**DOI:** 10.1371/journal.pone.0095343

**Published:** 2014-04-16

**Authors:** Xiao-Ping Chen, Wei-Feng Chen, Da-Wei Wang

**Affiliations:** College of Biological and Environmental Engineering, Zhejiang University of Technology, Hangzhou, China; Georgia Regents University, United States of America

## Abstract

Prenatal organophosphate exposure elicits long-term brain cytoarchitecture and cognitive function impairments, but the mechanism underlying the onset and development of neural progenitors remain largely unclear. Using precise positioned brain slices, we observed an alternated cleavage plane bias that emerged in the mitotic neural progenitors of embryonal neocortex with diazinion (DZN) and chlorpyrifos (CPF) pretreatment. In comparison with the control, DZN and CPF treatment induced decrease of vertical orientation, increase of oblique orientation, and increase of horizontal orientation. That is, the cleavage plane orientation bias had been rotated from vertical to horizontal after DZN and CPF treatment. Meanwhile, general morphology and mitotic index of the progenitors were unchanged. Acephate (ACP), another common organophosphate, had no significant effects on the cleavage plane orientation, cell morphology and mitotic index. These results represent direct evidence for the toxicity mechanism in onset multiplication of neural progenitors.

## Introduction

Developmental brain exposure to organophosphorus pesticides disturbs the neurogenesis procession and leaves long-term brain cytoarchitecture deficits and cognitive function impairments in adult animals [1], [2], [3], [4]. Such neurotoxicity can be induced by a variety of mechanisms, including selective inactivation of critical metabolic enzymes [5], [6], damage of chromosome of neural progenitors [7], [8], [9], [10], and disorder of content and turnover of neurotransmitters [11], [12]. For example, neuritic outgrowth in developing brain is impaired by chlorpyrifos (CPF) or diazinon (DZN) exposure with inhibition of choline acetyltransferase activity [13], nuclear condensation in oligodendrocyte progenitors is elicited by CPF co-incubation with increase of caspase 3/7 activity and heme oxygenase-1 expression [9], and depression behaviors in CPF-pretreated animals are dependence on abnormalities of serotonergic system [14]. Interest, early-prenatal CPF exposure or late-prenatal CPF exposure induce distinct reaction patterns of dopamine content in offspring brain regions, and equivalent neonatal CPF exposure causes distinct cytoarchitecture dysmorphogenesis in the striatum, somatosensory cortex and septal nucleus. Thus, the organophosphate-elicited neurotoxicity is developmental stages selective and brain regions selective [15], [16].

Previous evidences suggest that the selective brain region abnormalities are caused by the selective neurotoxicity effects on the proliferation and differentiation procession of neural progenitors. For example, inhibition effect of DNA synthesis in CPF treated C6 cells at the undifferentiated stage is much higher than that at the differentiated stage [17]. CPF or chlorpyrifos oxon (CPO) inhibits the axon outgrowth but enhances the dendrite growth in the superior cervical ganglia neuron [18]. CPF targets PC 12 cells to differentiate into the catecholaminergic phenotype, but inhibits the cells to differentiate into the cholinergic phenotype [19]. Moreover, in vivo studies showed that CPF-induced neurotoxicity affects gliotype cells to a greater extent than neuronotypic cells, with the maximum effects at the peak period of gliogenesis and glial cell differentiation [20]. However, there still lack of evidence that whether the toxicity involve the onset multiplication of neural progenitors.

Apical progenitors (APs) are the neurogenesis onset cells which locate at the apical surface of ventricular zone in embryo neocortex. APs perform two types of division: symmetric division for self-renew and asymmetric division for differentiation, which direct the two developmental fates in a single division [21], [22]. Cleavage plane orientation, the physical barrier of plasma contents, directs symmetric or asymmetric distribution of fate determinants in daughter cells and represents a hallmark of division types in APs [23], [24].

This study was designed to elucidate the direct effects of organophosphates on the cleavage plane orientation of neural progenitors. CPF, DZN and acephate (ACP), three common organophosphates, were treated in subtoxic dosages to the dams during E7.5–11.5, the cleavage plane orientations and the general morphology were measured at E16 in offspring neocortex APs. Our studies represent new exploration in the division procession of neural progenitor toxicity.

## Results

### Effects of Three Pesticides on General Cytoarchitecture of Apical Progenitors

**Figure 1:**
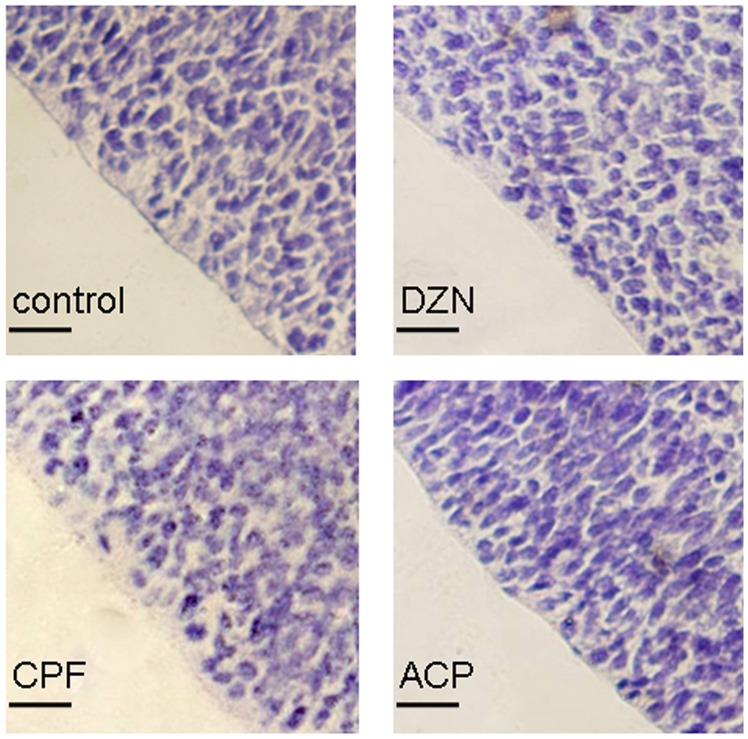
Effects of three pesticides on architecture of apical surface in E16 neocortex. Images of apical progenitors in E16 neocortex pretreated with diazinon (DZN, 2 mg/kg), chlorpyrifos (CPF, 2 mg/kg) or acephate (ACP, 50 mg/kg). The progenitors arranged radially along the ventricular margin with closely packed nuclei. Scale bar: 20 µM.

To examine the basic toxicity of three pesticides at the defined dosages, we first observed the general cytoarchitecture of APs on E16 neocortex slices. Apical progenitors at E16 were long and slender cells, radial arranged along the ventricular margin with closely packed nuclei (Fig. 1A). After pretreated with DZN, CPF and ACP during 7.5–11.5, the general cytoarchitecture of E16 APs had no obvious changes. The cells arranged in smooth line, the nuclei packed as the same density as in the control group, the intercellular spaces were in the normal range, and pathological changes like apoptosis bodies or vacuolus were not found in the APs cytoplasm (Fig. 1B, C, D).

### Effects of Three Pesticides on Mitotic Index of Apical Progenitors

**Figure 2:**
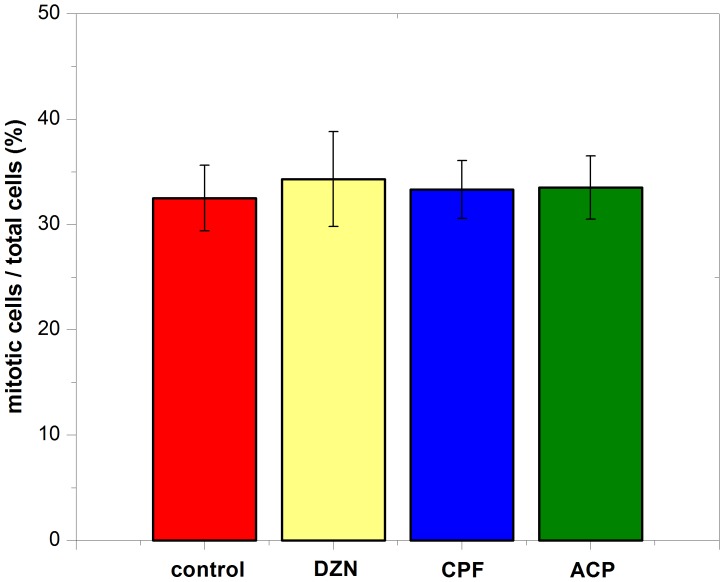
Effects of three pesticides on mitotic index of apical progenitors in E16 neocortex. Apical progenitors with mitotic figures were counted in three pesticides and control group, mitotic index was defined as the mitotic cell number/total cell number. More than 2000–3000 of cells was counted for each group. Data are represent as Mean ± SEM. N = 7 embryos from individual dams. No significance was observed at each group (P>0.05). Diazinon: DZN; Chlorpyrifos: CPF; Acephate: ACP.

To evaluate the effects of three pesticides on APs division procession, we determined the cell number in the APs with mitotic figures. More than 2000–3000 of cells was counted for each group, and the percentage of mitotic cells and mitotic index in all APs was calculated. Similar to previous studies [25], [26], mitotic index was 32.4±3.11% for control group. Respectively, mitotic index in DZN, CPF and ACP groups was 33.4±4.51%, 33.0±2.75%, and 33.2±3.01%. There was no statistics significance in all groups (P>0.05). (Fig. 2).

### Effects of Three Pesticides on Cleavage Plane of Apical Progenitors

Cleavage plane orientation was then been determined in E16 APs. Control group displayed 79.4±1.06% of vertical orientation, 14.7±0.73% of oblique orientation, and 5.4±0.53% of horizontal orientation. Respectively, the orientation index of DZN group and CPF group was 74.0±1.15% and 72.7±0.76% of vertical orientation, 17.8±0.94% and 21.4±1.12% of oblique orientation, 8.0±0.65% and 5.6±0.59% of horizontal orientation. Comparing to the control, there was significant decrease in vertical orientation of DZN and CPF group (P<0.05 and P<0.01), significant increase in oblique orientation of DZN and CPF group (P<0.05 and P<0.01), and significant increase in horizontal orientation of DZN group (P<0.01). These results indicated that DZN and CPF pretreatment rotate the orientation bias of APs from vertical to horizontal.

**Figure 3:**
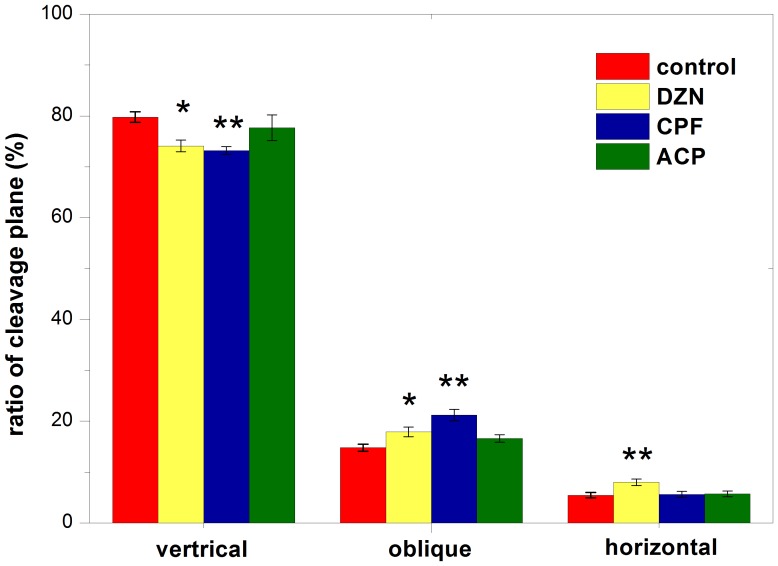
Effects of three pesticides on cleavage plane of apical progenitors in E16 neocortex. Cleavage plane orientation of apical progenitors was determined by measuring the angle of cleavage plane to the ventricle surface. Only cells in metaphase and anaphase phase were counted. Mitotic cells were divided into three types according the angle of cleavage plane plane: vertical (60–90°), oblique (30–60°), and horizontal (0–30°). (A) Effects of three pesticides on vertical orientation; (B) Effects of three pesticides on oblique orientation; (C) Effects of three pesticides on horizontal orientation. Data are represent as Mean ± SEM. N = 7 embryos from individual dams. *P<0.05; **P<0.01 for pesticides vs. control. Diazinon: DZN; Chlorpyrifos: CPF; Acephate: ACP.

In contrast, ACP pretreatment showed no significant effects on APs cleavage plane. There was 77.6±2.52% of vertical orientation, 16.6±0.73% of oblique orientation, and 5.7±0.57% of horizontal orientation in ACP group. No significant difference was found in all orientations comparing to the control group (P>0.05) (Fig. 3).

## Discussion

The occurrence and maintenance of the pesticides elicited neurotoxicity involve a variety of mechanism in proliferation and differentiation regulation at neural progenitors. In this study, we have performed a new research at the procession of cellular division, focusing on the morphology characteristics of the spindle mitotic figure.

There are three essential findings in our study. First, vertical cleavage plane could be decreased by two of three pesticides pretreatment, while oblique and horizontal cleavage plane could be increased in the meantime; Second, DZN and CPF exposure did not decrease the APs mitotic cells number; Finally, the two pesticides exposure at the defined dosages did not cause apoptosis and vacuolar degeneration of the progenitors. These results represented the cleavage plane abnormalities when no obvious basic pathology changes were observed in pesticides pretreated neural progenitors.

Roy et al [27] first reported the unnatural horizontal mitotic figures which usually not existing in E11.5 neuroepithelium, when incubating of E9.5 embryo of rat for 48 hrs in 0.5–50 µg/ml CPF solution. The abnormalities of cleavage plane were observed associated with the cell shrinkage and apoptosis. Prendergast et al [28] showed that pesticides treatment make propidium iodide incorporated into the progenitors spindle structure. These results suggest that organophosphorus pesticides have potent to direct impair the mitotic spindle and elicit division abnormalities. However, our data showed that altered cleavage plane orientation arise at the pesticides dosages without basic morphological abnormalities, which indicate the mitotic figure abnormalities as an early and sensitive hallmark of pesticides neurotoxicity.

Acetylcholinesterase (AChE) is commonly considered as the primary target of organophosphorus pesticides, which catabolyze neurotransmitter acetylcholine and play critical functions in cholinergic synaptic transmission [29]. Erythrocyte AChE activity is traditional toxicity endpoint of organophosphorus pesticides and standard point to establish the safety application doses [30]. Previously studies showed that CPF or DZN revealed developmental neurotoxicity at dose above the threshold of AChE inhibition, however, application of muscarinic receptors or nicotinic receptor antagonists showed little effect on the toxicity, indicating independent mechanism from the cholinergic functions [13], [29].

Several evidences suggested that the cleavage plane orientation may be determined by microtubules rearranged mechanism, for example, α-and β- tubulin dimmer assembling, microtubule associated protein bundling, and kinesin motor proteins driving [31], [32]. It was proved that microtubules polymerization can be disrupted by CPF and chlorpyrifos oxon (CPO) exposure, when covalently modified at the tyrosine sites of purified bovine tubulin and MAP-rich tubulin [33], [34]. Reduced MAP-2 immunoreactivity and injured pyramidal cells were found in cultured organotypic slices of hippocampus with CPO exposure [28]. Finally, in vitro experiments showed CPF and CPO exposure inhibit the microtubulin mobility directed by molecule motor kinesin [35]. Thus, microtubule mechanism may involve in the pesticide-induced mitotic figure abnormalites.

## Materials and Methods

### Animal and Treatment

ICR mice, weight of 28∼32 g and aged at 8–10 weeks, were purchased from the experimental animal centre in Zhejiang province. Male and female couples were housed in breeding cages with free access to food and water and a 12/12 dark-light cycle. Vaginal plugs were checked twice a day at 9∶00 am and 9∶00 pm to determine the exact time of embryo day 0 (E0). All the experimental procedures were conducted with the approval by the Institutional Animal Ethics Committee of the Research Center of Laboratory Animal Science of Zhejiang Chinese Medical University(Permit Number are SYXK (Zhe) 2008-0116) and carried out in accordance with *the Guide for the Care and Use of Laboratory animals* issued by the National Institutes of Health.

The pregnant female mice were randomly classified into four groups with seven dams for each treatment group. Chlorpyrifos (CPF, 99.1% purity) was obtained from Xinnong Chemical Company, Zhejiang. Diazizon (DZN, 98.3% purity) and acephate (ACP, 97% purity) were purchased from Sigma Company. CPF, DZN and ACP were dissolved in dimethyl sulfoxide at concentrations of 1 mg/ml, 1 mg/ml and 25 mg/ml. From E7.5 to E11.5, dams were received daily subcutaneous injections of 2 mg/kg.bw DZN, 2 mg/kg.bw CPF and 50 mg/kg.bw ACP, while the control group was injected with an equal volume of dimethyl sulfoxide (volume range from 0.056 ml to 0.064 ml according to the maternal body weight).

### Tissue Fixation and Histochemistry

On E16, dams were sacrificed, embryo brains were isolated and fixed in 4% paraformaldehyde for 24 hour. After dehydrated with gradients alcohol and cleared with xylene, the samples were embedded in paraffin. The wax block was adjusted to take a precise coronary orientation, then cut into continuous 4-µm sections. The sections were de-crumpled in 45°C water and put onto glass slices. The slices were dried in 65°C over night, de-paraffinized with gradients alcohol, cleared with xylene, then stained with hematoxylin for 15 min and counterstained with eosin for 30 sec.

### Cytoarchitecture

Maximum cross-section of lateral ventricle was used as the hallmark to maintain the slices at same level. Cytoarchitecture of apical surface of E16 neocortex ventricle wall was observed and distinguished under Olympus IX71 microscope. Apical progenitors were defined as the cells lying along the surface of ventricular zone. General cell morphology was observed under high magnification, and mitotic index is defined as the mitotic cell number/total cell number.

To reduce any individual differences in groups, all the results are performed in a double-blind way. Seven slices in each group was taken from seven different embryos with individual dams.

### Cleavage Plane Orientation

Mitotic figures in apical surface of ventricular zone were recognized and analyzed. Cleavage plane orientation was determined by measuring the angle of cleavage plane to the ventricle surface. Only cells in metaphase and anaphase phase were counted. Mitotic cells were divided into three types according the angle of cleavage plane plane: horizontal (0–30°), oblique (30–60°), and vertical (60–90°). Each type of orientation was counted separately and calculated as the ratio of total counted mitotic cells.

### Data Analysis

Data are presented as means plus standard error (Mean ± SEM). SPSS 17.0 software was used to analysis treatment and control groups with single-factor analysis of variance (ANOVA, one-way analysis of variance). Significance was conducted and expressed as P<0.05 and P<0.01.
